# Quality of life assessments in a cohort of Mozambican children with sickle cell disease

**DOI:** 10.11604/pamj.2020.36.343.24837

**Published:** 2020-08-25

**Authors:** Faiaz Issa, Brian Norman Dang, W Chris Buck, Sérgio Chicumbe, Nelsa Nicolau, Chana Virate, Naya Cassamo, Angelina Dias, Faizana Amodo

**Affiliations:** 1 Hospital Central de Maputo, Maputo, Mozambique,; 2 University of California Los Angeles, David Geffen School of Medicine, California Los Angeles, USA,; 3 Instituto Nacional de Saúde, Maputo, Mozambique

**Keywords:** Sickle cell disease, quality of life, hydroxyurea, children, pediatrics, Mozambique

## Abstract

**Introduction:**

sickle cell disease (SCD) has significant pediatric morbidity and mortality in sub-Saharan Africa, where access to therapies such as hydroxyurea and opioids is often limited. Poor disease control and Pain management adversely affects the well-being and mental health of affected children. Questionnaires have been utilized in other regions to report the quality of life (QOL) in children with SCD, but assessments from Africa are lacking.

**Methods:**

children age 2-14 years with SCD presenting for routine outpatient consultations at Hospital Central de Maputo from June-August 2017 were offered participation. After informed consent, the Pediatric QOL Inventory (PedsQL) SCD Module was administered to all caregivers and children > 5 years. Responses were scored from 0-100, with higher scores representing better QOL.

**Results:**

a total of 14 children were included, with six (43%), four (29%), two (14%), and two (14%) from the age groups of 2-4, 5-7, 8-12, and 13-14 years, respectively. Mean overall patient QOL was 65.3 and 56.0 in child and caregiver respondents. In patients > 5 years, the difference in mean overall QOL for those on/not on hydroxyurea was 0.6 (66.5-64.9) in child respondents and 15.8 (68.4-52.6) in caregiver respondents. Domains related to worry/emotions and communication scored lower in QOL than Pain-related domains for both patient and caregiver respondents.

**Conclusion:**

SCD has a negative impact on QOL as reported by this cohort of Mozambican pediatric patients and caregivers, with Pain being less of a concern than emotional and interpersonal issues. A comprehensive, child-focused care approach with robust psychosocial support is needed.

## Introduction

Sickle cell disease (SCD) is a group of inherited hematological disorders characterized by vaso-occlusive Pain crises, multiorgan infarction, chronic anemia, and reduced life expectancy [1]. It is estimated that the majority of sickle cell births occur in low-resourced countries, such that sub-Saharan Africa alone contributes to more than 200,000 new infants diagnoses each year [2]. Despite the high prevalence of sickle cell disorders (up to 3% of births in some parts of sub-Saharan Africa), with estimated mortality rates around 50-90%, this group of hemoglobinopathies remains largely neglected [3, 4]. Currently, SCD is not recognized as one of the top five leading causes of global mortality in children less than 5 years of age; however, many infants are undiagnosed at time of death [5, 6]. Thus, the true burden of SCD is vastly underappreciated due to poor diagnostic tests and the absence of a standardized protocol for newborn screening [2, 6].

The hallmark of SCD is the reoccurrence of vaso-occlusive episodes that contributes to acute and chronic Pain [7]. Hydroxyurea is a disease modifying drug that has proven efficacy in reducing the frequency of Pain crises in patients with SCD [8]. Unfortunately, this agent is minimally utilized in resource-limited regions due to cost, unavailability of pediatric formulations, and inexperience among health care providers [9, 10]. This burden of resource limitation is also evident in the barriers to Pain management that include cultural beliefs, inadequate training, and decreased availability and utilization of opioid therapy [11-13].

Despite the growth of research on the methods of screening, prophylaxis, and treatment of children with SCD, less emphasis has been placed on the psychosocial impact of this condition [14]. Current studies demonstrate that the multifaceted effects of SCD on one´s mental health, and the chronic Pain associated with this condition, negatively impact psychologic and physical quality of life [15, 16]. Further, those of low socioeconomic status are at increased vulnerability to the negative social determinants of SCD [17]. Thus, studies to explore the psychosocial effects of SCD in resource-limited populations are urgently needed.

Various quality of life measures exist to assess the psychosocial consequences of SCD [18]. The Pediatric Quality of Life Inventory (PedsQL) is a standardized instrument that measures health-related quality of life in pediatric populations, and disease-specific modules have been created [19]. The PedsQL SCD Module is a validated tool that has been utilized in children with SCD in the United States, Jamaica, Brazil, and Oman [20-24]. However, no studies from Africa were identified from our literature search. In this study, we assessed the impact of SCD in a cohort of Mozambican children using the PedsQL SCD tool.

## Methods

**Setting:** this study took place at Hospital Central de Maputo (HCM), Mozambique´s largest and highest-level referral hospital. HCM is also the principal academic hospital in the country. The Pediatrics Department has a Hematology Oncology division with inpatient and outpatient services, led by a pediatric subspecialist. Routine hematology labs are available in addition to hemoglobin electrophoresis for SCD diagnosis. Child-friendly 200mg hydroxyurea tablets only became available in 2019. Parenteral narcotics are available in the inpatient setting for the treatment of Pain, but there is no patient - controlled analgesia. Oral opioids are not used for the ambulatory management of Pain.

**Patients:** patients with SCD who presented to the pediatric hematology outpatient service at HCM for regularly scheduled follow up appointments during the time period of June 1 - Aug 31, 2017 were recruited. Inclusion criteria were: confirmed SCD diagnosis per hemoglobin electrophoresis, > 2 and < 15 years of age, and clinic enrollment between January 1, 2011 and July 1, 2016 with at least one year of follow-up time.

**Data collection:** after informed consent and assent, when applicable, quality of life (QOL) was assessed via the PedsQL SCD Module, which is available with an official Portuguese language translation [19]. A trained psychologist administered an age-specific PedsQL SCD questionnaire to patients about their QOL and caregivers about their child´s QOL (Teen 13-14 years, Child 8-12 years, Young Child 5-7 years, and Toddler 2-4 years). For the toddler age group, the questionnaire was only administered to caregivers; for all other age groups, both patients and caregivers were surveyed.

The questionnaire includes QOL domains that pertain to Pain and hurt, Pain impact, Pain management and control, worrying (two domains), emotions, treatment, and communication (two domains). The scoring of the questionnaire utilizes a 5-point Likert scale (0 = never a problem to 4 = almost always a problem), with exception of the young child report that utilizes a “not at all,” “sometimes,” and “a lot” grading schematic represented by face scales that correspond to scores of 0, 2, and 4 respectively. Scoring procedures were executed in accordance to the scoring protocol supplied by PedsQL [25].

Questionnaire responses were transcribed into a Microsoft Excel® database. Clinical and demographic data including laboratory results and treatment regimens were extracted from medical charts and also entered into the same database.

**Analysis:** following standard PedsQL methodology, scores were averaged per domain, and then reverse scored and linearly transformed to a 0-100 scale with higher scores representing higher QOL. For each patient and caregiver respondent, scores were then averaged across all domains to establish a single composite total score. Means were calculated for caregiver and patient responses. Questionnaire domains were grouped into three categories for sub-analysis; 1) Pain and Treatment (includes Pain and Hurt, Pain Impact, Pain Management and Control, and Treatment), 2) Worry and Emotion (includes Worry 1, Worry 2, and Emotions), and 3) Communication (includes Communication 1 and Communication 2). Due to a relatively small sample size, purely descriptive statistics (differences between means) were used to compare mean scores according to demographic and clinical predictive variables.

**Ethical considerations:** this study was approved by the Scientific Directorate of HCM and received final bioethical approval from the Mozambique National Bioethics Committee (53/CNBS/2017). Informed consent was obtained from all caregivers and informed assent from all patients 13-14 years of age. Caregivers served as the consenting parties for all children. Since potential hazards from completing the study questionnaire included emotional distress and potential disagreements between patients and their caregivers on certain issues, the surveys were administered by trained clinical psychologists and child and caregiver questionnaires were administered separately.

## Results

**Patient characteristics:** this study includes 14 patients with SCD (Table 1). The age distribution was six (43%), four (29%), two (14%), and two (14%) patients from the age groups of 2-4, 5-7, 8-12, and 13-14 years, respectively. Mean hemoglobin was 7.2 g/dL and all patients were HIV negative. All patients had diagnostic confirmation by hemoglobin electrophoresis, whereby 13 (93%) had HbSS and one (7%) had HbSD. Of the patients with HbSS, the mean HbS was 81.7%. Three (21%) of the patients were on hydroxyurea.

**Table 1:** patient characteristics

No. of patients	14
**Age (years)**	
2-4	6 (43%)
5-7	4 (29%)
8-12	2 (14%)
13-14	2 (14%)
**Sex**	
Female	7 (50%)
Male	7 (50%)
**Hgb Electrophoresis**	
HbSS	13 (93%)
HbSD	1 (7%)
**HIV Positive**	0 (0%)
**Hydroxyurea treatment**	3 (21%)
**Hgb level (mean g/dL)**	7.2

Hgb - hemoglobin; HIV - human immunodeficiency virus

### PedsQL SCD Module Results

**Figure 1:**
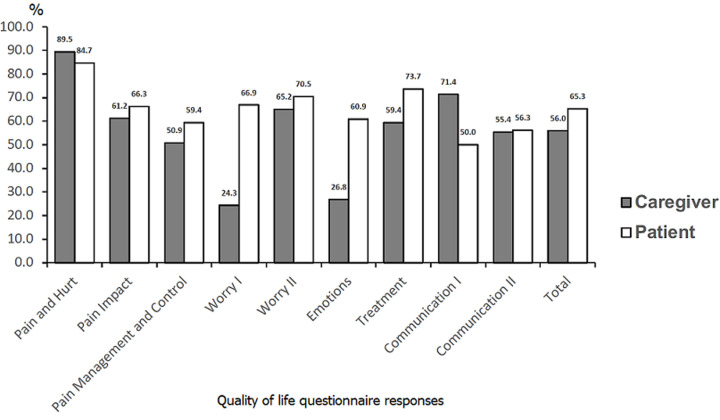
quality of life questionnaire responses, by caregivers and patients; caregivers of patients from all ages were included, while only patients > 5 years old were included

**Total score:** comparisons were made between the responses of SCD patients and their caregivers (Figure 1). Caregivers of patients from all age groups were included, however patients 2-4 years of age were excluded since they did not complete their own questionnaires. The total mean scores from the patients were higher than the total mean scores from the caregivers (65.3 versus 56.0, difference of 9.3). When disaggregated by age, children´s mean overall QOL was consistently higher than that reported by their caregivers for all age groups, including 5-7 years (69.1 versus 64.1, difference of 5.0), 8-12 years (60.9 versus 45.9, difference of 15.0), and 13-14 years (62.1 versus 59.1, difference of 3.0).

Comparisons were made between the responses of SCD patients and caregivers based on hydroxyurea treatment, excluding patients 2-4 years of age. Children on hydroxyurea reported little difference in mean total QOL compared to those not on treatment (66.5 versus 64.9, difference of 1.6). However, caregivers reported a much larger perceived benefit of hydroxyurea treatment (68.4 versus 52.6, difference of 15.7).

**Figure 2:**
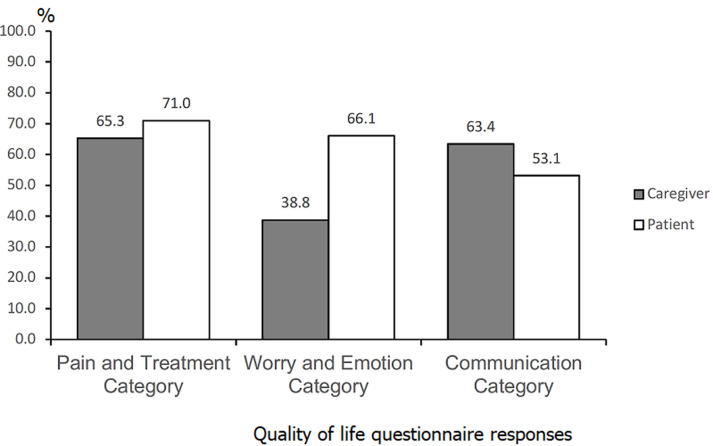
quality of life questionnaire responses, grouped domains, by caregivers and patients; caregivers of patients from all ages were included, while only patients > 5 years old were included

**Domain sub-analysis:** mean scores per grouped domain categories were also compared by respondent (Figure 2). For both children and caregivers, the Pain and Treatment category had the highest mean QOL (71.0 versus 65.3, difference of 5.7). For the Worry and Emotion category, children reported a much higher mean QOL than their caregivers (66.1 vs 38.8, difference of 27.4). For the Communication category, children reported a lower mean QOL than their caregivers (53.1 versus 63.4, difference of -10.3).

These general trends persisted on age-disaggregated analysis with some minor variations. The Pain and Treatment category scores were among the highest for both children and caregivers across all ages. The Worry and Emotion category scores were consistently higher for children across all age groups compared with the corresponding caregivers. The Communication category scores were much lower for children compared with their caregivers in the 5-7 year (62.5 versus 83.3, difference of -20.8) and 13-14 year (50.0 versus 79.2, difference of -29.2) age groups, but higher for children in the 8-12 year (37.5 versus 29.2, difference of 8.3) age group.

**Figure 3:**
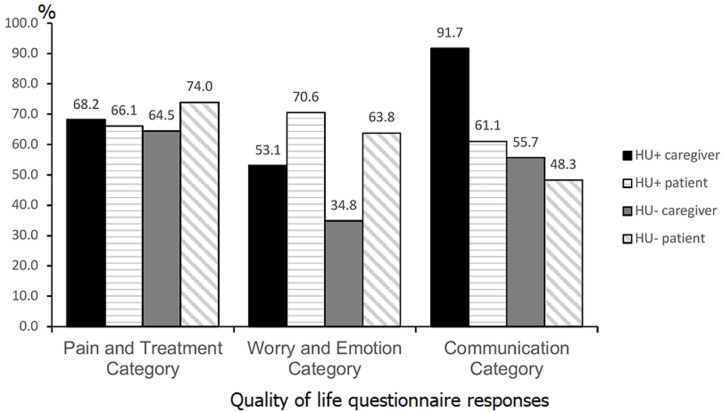
quality of life questionnaire responses, caregivers and patients grouped domains, by hydroxyurea; caregivers of patients from all ages were included, while only patients > 5 years old were included

Mean scores per grouped domain categories were also compared between respondents according to hydroxyurea treatment (Figure 3). In children, the Communication category had the lowest mean QOL for those receiving and those not receiving treatment (61.1 and 48.3, respectively). For caregivers, the Worry and Emotions category had the lowest mean QOL for children receiving and not receiving hydroxyurea (53.1 and 34.8, respectively).

## Discussion

To our knowledge, this is the first reported study to utilize the PedsQL SCD Module to assess the impact of SCD on QOL from the African continent. Our results show that SCD has a significant negative impact on QOL from the patients´ perspective, with a mean total score of 65.3 out of a maximum of 100. These findings are similar to published reports from other global pediatric cohorts that used the PedsQL SCD tool in Jamaica (73.6 in adolescents) and Brazil (60.7 in children and adolescents) [22, 23]. Of note, a study from Oman reported much lower overall QOL (33.2 - 42.4) [24].

Our cohort had relatively similar QOL compared with two studies from the United States that reported total scores of 65.8 and 73.6 for child respondents [21, 22]. Given what is known about the association between socioeconomic status and poor SCD disease outcomes, in addition to the substantial differences in the capacity of the health care systems between the United States and Mozambique, we anticipated much lower relative QOL scores in our study [12, 17]. While the purpose of this study was not to assess the validity of the PedsQL SCD Module in an African setting, our findings suggest that comparisons of absolute numerical results among studies from various regions should be made with caution.

Another surprising finding from this study was that the highest QOL scores were observed in the Pain and Treatment category for both patients and caregivers. It is well established that Pain is undertreated and inadequately evaluated in most sub-Saharan African settings [12, 13, 26, 27]. The barriers to pediatric Pain management in this region include decreased availability of analgesic medications, inadequate healthcare worker training, and opiate regulations that disproportionately affect low-resourced countries [11]. Without oral opioids and patient-controlled analgesia for Pain management in the outpatient and inpatient settings in Mozambique, we had anticipated that QOL scores would be relatively lower for the Pain and Treatment category compared to the Worry/Emotion and Communication categories. Thus, local cultural norms related to the perception of Pain, communication about Pain, and expectations about feasible Pain control may have influenced the results in our study [11].

While analgesics are important for SCD Pain management, hydroxyurea is the only drug that effectively reduces the frequency of Painful vaso-occlusive episodes and prolongs survival in patients with SCD [28-30]. Further, recent results from the REACH trial have demonstrated the feasibility, safety, and benefits of hydroxyurea for children with SCD in sub-Saharan Africa, with reduced incidence of Pain crises, malaria, transfusions, and death [31]. In terms of QOL, hydroxyurea has also been found to improve social functioning, reduce symptoms of Pain and tension, and improve overall health-related quality of life [32, 33]. In our study, caregivers of children on hydroxyurea reported higher QOL for every category group compared to caregivers of children not on hydroxyurea. On the other hand, children on hydroxyurea reported higher QOL in the Worry/Emotion and Communication categories, but not in Pain and Treatment. These unexpected findings in the child responses may be a result of selection bias, as patients with more severe, symptomatic disease were more likely to be initiated on hydroxyurea at the time of this study.

The lack of agreement between caregiver and patient responses to the PedsQL SCD Module was also noteworthy in our cohort, and has been noted in other studies using the tool [34]. Caregivers rated overall QOL 9.3 points lower than children (56.0 vs 65.3), a difference of 16.6%. In the grouped domain sub-analysis, the Worry/Emotion category was the area of lowest reported QOL for caregivers (38.8 vs. 66.1 in children, difference of 70.4%), while the Communication category had the lowest reported QOL for children (53.1 vs 63.4 in caregivers, difference of 19.4%). This discrepancy between child and caregiver results in all age groups highlights the need for customized psychosocial support for both children with SCD and caregivers that prioritize issues relating to communication and worry, respectively. Regardless of the child´s age, efforts must be made to provide child-center care and engage the patient in the history and treatment discussions in all consultations, as their caregivers´ assessments may not match their own. For caregivers of children with SCD, optimizing parental support has been found to decrease the patient´s depression and improve overall QOL [35].

This study is not without limitations. We had a small sample size and were unable to extend recruitment beyond the mentioned study period to include more patients. As such, we used purely descriptive statistics and did not have the power needed to determine if differences had statistical significance. The level of care available at HCM is far beyond what is available in most other parts of Mozambique, which may limit the generalizability of our findings. In addition, as with any questionnaire, there was a possibility of responder bias, but we tried to mitigate this possibility by having psychologists who did not regularly participate in the SCD clinic administer the PedsQL SCD Module as opposed to regular clinical staff. And, as mentioned previously, there was likely some selection bias in that patients on hydroxyurea therapy were more likely to have advanced disease.

Despite these limitations, this study helps fill an important knowledge gap in the literature regarding the psychosocial impact of SCD in African children. These results can be used to advocate for comprehensive medical and psychosocial care of children with SCD and have already had a general stimulatory effect in terms of quality improvement at HCM, with an additional three patients from the cohort initiated on hydroxyurea in the year following completion of the PedsQL SCD questionnaire. We plan to conduct a similar evaluation in the future with children admitted to the wards with SCD to better understand the patient and caregiver experience, especially in regards to Pain and Pain management.

## Conclusion

SCD has a negative impact on quality of life as reported in this cohort of Mozambican pediatric patients and their caregivers, with Pain being less of a concern than emotional and interpersonal issues. A comprehensive, child-focused care approach with robust psychosocial support, in addition to evidence-based medical therapies that include hydroxyurea and opioids for Pain crises, is needed to improve overall mental and physical health outcomes.

### What is known about this topic

- Sickle cell disease can have a significant impact on the physical health and psychosocial wellbeing of affected children and adolescents;
- Validated questionnaires such as the PedsQL Sickle Cell Disease tool have been used in other parts of the world to assess quality of life in affected children.

### What this study adds

- Using the PedsQL tool, Mozambican pediatric patient and caregiver respondents generally reported lower quality of life in emotional and communication domains, than in Pain-related domains;
- Quality of life assessments often differ between children and their caregivers;
- A child-focused, comprehensive care approach with psychosocial support is needed for children and adolescents in care for sickle cell disease.
